# Prosthodontic Management of Congenitally Missing and Peg-Shaped Maxillary Lateral Incisors: A Case Report

**DOI:** 10.7759/cureus.83651

**Published:** 2025-05-07

**Authors:** Madhurima Sharma, Rohit Nandan, Rohit Sharma, Shalabh Kumar, Akash Gopi, Raina Agarwal, Samra Ashraf, Srijita Mahata, Papan Barman

**Affiliations:** 1 Prosthodontics and Crown and Bridge, Teerthanker Mahaveer Dental College and Research Centre, Moradabad, IND; 2 Conservative Dentistry and Endodontics, Teerthanker Mahaveer Dental College and Research Centre, Moradabad, IND; 3 Periodontology, Babu Banarasi Das College of Dental Sciences, Lucknow, IND

**Keywords:** aesthetics, cantilever bridge, peg lateral incisor, restoration, zirconia

## Abstract

This case report describes the prosthetic rehabilitation of a 24-year-old female patient who had aesthetic concerns due to implant failure in the right maxillary lateral incisor and peg-shaped left maxillary lateral incisor sites. The treatment plan included fabrication of a canine-supported zirconia cantilever prosthesis for the right incisor and a zirconia crown to restore the peg-shaped left incisor. A minimally invasive approach was employed, including abutment preparation, computer-aided design/computer-aided manufacturing (CAD/CAM) design, and precision milling of the zirconia restoration. The final restoration was cemented with resin cement. The result was the successful restoration of symmetry, functionality, and aesthetics in the maxillary anterior teeth, significantly improving the patient's smile and self-confidence.

## Introduction

The absence of maxillary lateral incisors and peg-shaped lateral incisors are common dental anomalies that present functional and aesthetic challenges. Implant-supported prostheses are often the preferred treatment; however, complications such as implant failure necessitate alternative solutions [1].

To address these challenges, this report describes a conservative approach using a zirconia cantilever prosthesis. Implant-supported prostheses have long been the favoured method for replacing missing lateral incisors because of their durability, aesthetics, and ability to maintain adjacent teeth. However, implant therapy has limits. Insufficient bone volume, peri-implantitis, and implant failure can all jeopardize the long-term success of this treatment technique. Alternative options must be investigated for patients who experience implant failure or are deemed unsuitable for reimplantation due to structural or systemic restrictions [2,3].

In such cases, a cautious strategy might lead to predictable and effective results. This case report describes the use of a canine-supported zirconia cantilever prosthesis as an alternative to standard implant therapy for a lost lateral incisor. Zirconia has exceptional esthetic and biomechanical qualities, making it an excellent choice for anterior restorations [4,5]. Furthermore, a peg-shaped lateral incisor was restored using zirconia crowns to improve the aesthetics.

This approach not only solves the functional and aesthetic issues associated with these defects but also reduces the need for additional surgical procedures, lowering patient morbidity. The example emphasizes the significance of tailored treatment planning, as well as the use of current materials and techniques, to provide long-term solutions. Conservative and inventive treatments, which focus on patient-specific demands, can restore oral function and aesthetics, even in difficult instances involving implant failures or congenital dental defects.

## Case presentation

**Figure 1:**
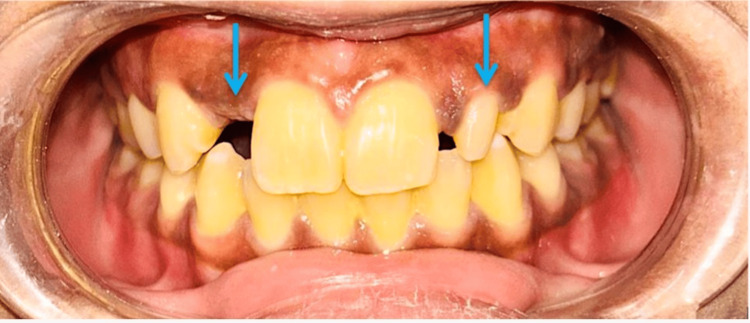
Pre-operative view showing the failed implant site of the missing maxillary right lateral incisor and maxillary left peg lateral incisor

A 24-year-old female arrived in the Department of Prosthodontics with the primary complaint of discontent with the aesthetics of her upper front teeth. She had previously had an implant placed in the maxillary right lateral incisor site, which failed within one year after placement due to peri-implantitis. In addition, the patient expressed concern about the size and shape of her maxillary left lateral incisor (Figure 1).

The patient was provided with multiple treatment options, such as replacement of the implant, a removable partial denture, and a conventional three-unit bridge (fixed partial denture) in place of a cantilever bridge. Out of which, she decided to go for a zirconia cantilever bridge and zirconia crown.

Treatment plan

We decided to fabricate a canine-supported zirconia cantilever prosthesis to replace the right lateral incisor and for the restoration of the peg-shaped left lateral incisor with zirconia crown to harmonize tooth proportions.

Treatment procedure

**Figure 2:**
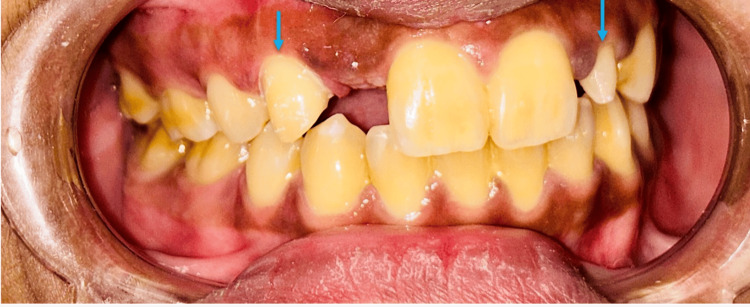
Prepared maxillary right canine for the zirconia cantilever-fixed partial denture to restore the maxillary right lateral incisor and the prepared maxillary left peg lateral for restoration via a zirconia crown

The treatment process began with the preparation of the maxillary right canine and peg lateral to serve as abutments for a zirconia cantilever-fixed partial denture and a zirconia crown, respectively (Figure 2).

Once the teeth were carefully shaped, a putty wash impression with Aquasil Ultra that is a poly vinyl siloxane impression material was made to capture precise details, ensuring a well-adapted prosthesis. This impression, along with the patient’s bite records and maxillary and mandibular casts, was then sent to the laboratory for further processing.

**Figure 3:**
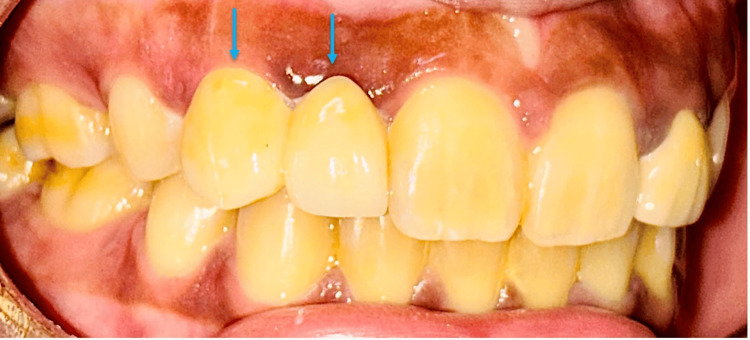
Cementation of the zirconia cantilever-fixed partial denture on the maxillary right canine with dual-cure resin cement to restore the right lateral incisor

Using CAD/CAM technology, the zirconia cantilever prosthesis and zirconia crown were digitally designed and milled, ensuring high precision and natural aesthetics. During the try-in phase, adjustments were made to achieve optimal fit and function before final cementation with dual-cure resin cement (Figure 3).

After the try-in of the zirconia cantilever, the crown was sandblasted with aluminium oxide and cleaned with alcohol and dried with air. The prepared tooth was cleaned with pumice paste, and then, according to the manufacturer's instructions, the dual-cure resin cement (RelyX Universal) was dispensed directly into the zirconia crown with an auto-mix syringe. The crown was seated onto the tooth firmly with finger pressure and then cured for one to two seconds. The excess cement was removed with a scaler and finally light cured for 20 seconds per surface.

**Figure 4:**
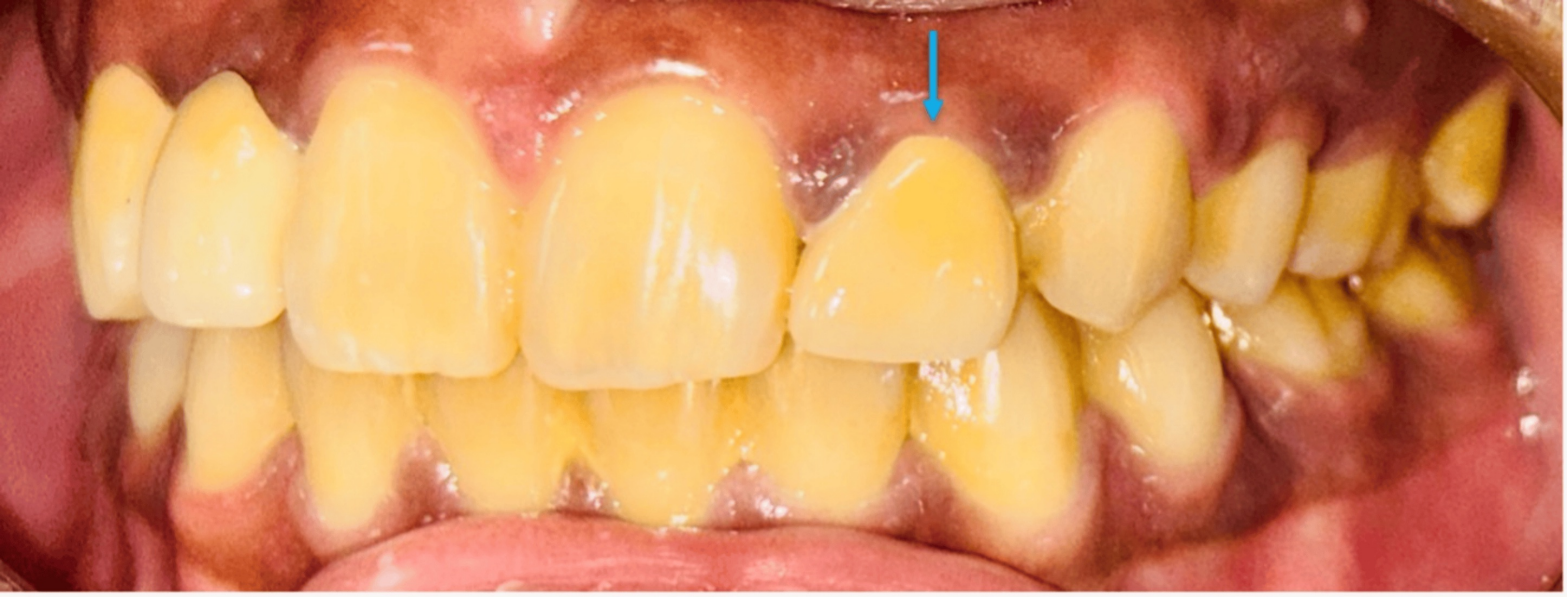
Cementation of the zirconia crown on the maxillary left peg lateral incisor with dual-cure resin cement to restore aesthetics

Simultaneously, the zirconia crown was placed to restore the peg lateral, ensuring a harmonious alignment with the adjacent dentition (Figure 4).

Outcome and follow-up

**Figure 5:**
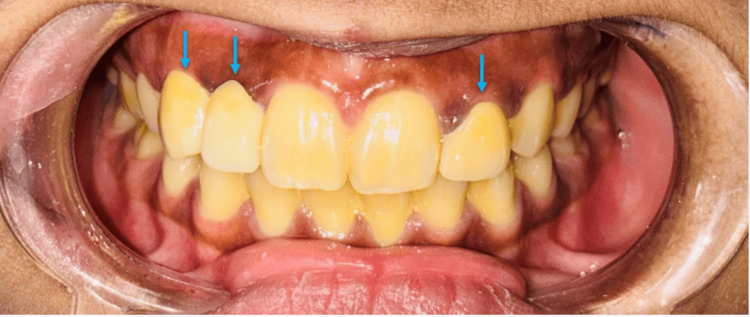
Post-operative view showing the final outcome of the zirconia cantilever-fixed partial denture and the zirconia crown for the restoration of aesthetics and function

The procedure was highly successful in restoring both function and aesthetics, resulting in anterior maxillary symmetry. The zirconia cantilever prosthesis provided a durable and lifelike replacement for the right and left maxillary lateral incisors, seamlessly integrating with the surrounding teeth (Figure 5).

The patient was extremely satisfied with the outcome, appreciating the enhanced smile, stability, and long-term reliability of the restorations.

Follow-up duration was one year, and patient follow up visits were in every six months. Treatment was successful in restoring both function and aesthetics.

## Discussion

Cantilever prostheses are a practical and reliable alternative in the event of implant failure, especially when supported by strong abutment teeth, such as canines, which provide good stability and load distribution. The report highlights the clinical effectiveness of zirconia's strength, biocompatibility, and aesthetics, as well as the reliability of cantilever prostheses supported by sturdy abutments, such as canines. Individualised treatment planning and delivery were found to be critical to addressing patient concerns and achieving long-term success.

This method reduces the need for future surgical procedures while ensuring functional and aesthetic rehabilitation. Zirconia, known for its high mechanical strength, biocompatibility, and aesthetic appeal, is an excellent material for rebuilding peg-shaped teeth and structurally impaired dentition. Its wear resistance, high fracture toughness, and natural translucency make it an excellent choice for anterior restorations, ensuring longevity and a lifelike appearance [6-8].

Kern et al. [8] examined 108 anterior zirconia ceramic cantilever resin-bonded fixed dental prosthesis (RBFDPs) over an average observation time of 7.7 years. The results showed a 10-year survival rate of 98.2% and a success rate of 92.0%, demonstrating that the material is durable and successful in anterior restorations [8].

Kallala et al. [5] stated that peg-shaped lateral incisors can be replaced using zirconia crowns to improve their shape and proportion, resulting in a harmonious and natural smile [5].

Horsch et al. [9] studied the survival and complication rates of all-ceramic and metal-ceramic implant-supported cantilever-fixed dental prosthesis (cFDPs). The retrospective research revealed positive results for all-ceramic cFDPs, bolstering the viability of zirconia-based restorations in complicated dental patients [9].

There's limited research available on how cantilever zirconia RBFDPs perform over extended periods when they are used to replace missing incisors. A recent study by Kern et al. [10] assessed how well these prostheses survive, stay attached, and succeed when they are used to replace missing incisors in both the upper and lower jaw. The research concluded that these cantilever zirconia RBFDPs offer a reliable treatment option that requires a minimal removal of healthy tooth structure side-wise, providing great clinical and aesthetic outcomes when replacing missing incisors [10].

Maximum aesthetics can be achieved with ceramic veneers, which provide a conservative treatment option for peg-shaped lateral incisors. The success of these ceramic veneers largely depends on proper case selection [11].

These studies underscore the importance of personalised, minimally invasive treatment planning in addressing anterior dental abnormalities. By leveraging advanced materials such as zirconia, clinicians can achieve durable, aesthetically pleasing, and clinically effective solutions for complex dental conditions.

The successful treatment not only restored the patient's smile but also increased faith in zirconia-based restorations as a long-lasting and aesthetically acceptable solution.

## Conclusions

This instance demonstrates the importance of personalized, least-invasive dental treatment in getting the best aesthetic and functional outcomes. The application of a canine-supported zirconia cantilever prosthesis for the failed implant site and a zirconia crown for the restoration of a peg-shaped lateral incisor displays zirconia's versatility and dependability in modern restorative dentistry. By addressing the patient's key concerns, improving the symmetry, proportion, and aesthetics of the front maxilla, this treatment demonstrates the value of a holistic and patient-centered approach. This case serves as a reminder that careful treatment planning and execution can transform challenges into opportunities for long-term success and patient satisfaction.
